# P15 Evaluation of the stability of temocillin in elastomeric infusion devices used for outpatient parenteral antimicrobial therapy in accordance with the requirements of the UK NHS Yellow Cover Document

**DOI:** 10.1093/jacamr/dlac004.014

**Published:** 2022-02-16

**Authors:** Fekade B. Sime, Steven C. Wallis, Conor Jamieson, Tim Hills, Mark Gilchrist, Mark Santillo, R. Andrew Seaton, Felicity Drummond, Jason A. Roberts

**Affiliations:** The University of Queensland Centre for Clinical Research, Brisbane, Australia; The University of Queensland Centre for Clinical Research, Brisbane, Australia; Pharmacy Department, Sandwell and West Birmingham NHS Trust, UK; Pharmacy Department and OPAT Service, Nottingham University Hospitals, Nottingham, UK; Department of Pharmacy/Infection, Imperial College Healthcare NHS Trust, London, UK; Torbay & South Devon NHS Foundation Trust, Torquay, UK; Department of Infectious Diseases, Queen Elizabeth University Hospital, Glasgow, UK; British Society for Antimicrobial Chemotherapy, Birmingham, UK; The University of Queensland Centre for Clinical Research, Brisbane, Australia

## Abstract

**Background:**

To assess the feasibility of its use in OPAT via continuous infusion, the stability of temocillin solutions at clinically relevant concentrations in two elastomeric infusion devices (B. Braun Medical Ltd Easypump® II LT 270-27- S and Spirit Ltd Medical Dosi-Fuser® L25915-250D1) was evaluated during 14 days of (2°C–8°C) fridge storage followed by 24 h exposure in-use temperature at 32°C, when reconstituted with 0.3% citrate buffer at pH 7.

**Methods:**

Stability testing was conducted in accordance with the standard protocols for deriving and assessment of stability of small molecule aseptic preparation as per the latest UK National Health Service Pharmaceutical Quality Assurance Committee Yellow Cover Document (YCD) requirements. A stability indicating assay method was developed with an ultra-HPLC (UHPLC) system using photodiode array detector. Temocillin concentrations corresponding to low (500 mg/240 mL), intermediate (4000 mg/240 mL) and high (6000 mg/240 mL) dose in triplicate devices were tested with duplicate samples at 11 timepoints during 14 days of fridge storage followed by 24 h in-use temperature exposure at 32°C.

**Results:**

A total of 396 samples were collected and assayed. The percentage of temocillin remaining after 14 days of fridge storage was greater than 97% in both devices and at all concentrations tested. During in-use temperature, 95% stability limit was achieved for 12 h for all doses and devices tested except for the high concentration in the Dosi-Fuser® device, which met this criterion for only 10 h of in-use temperature exposure.

**Conclusions:**

Temocillin reconstituted with 0.3% citrate buffer at pH 7 in elastomeric infusion devices can be stored in a fridge (2°C–8°C) for 2 weeks, meeting the YCD acceptance criteria of <5% degradation. Within the UK, the current data supports twice daily dosing of temocillin with <5% degradation at in-use temperature of 32°C for 12 h, except at high dose (6000 mg/240 mL) in the Dosi-Fuser® device, which must be limited to 10 h. The limits of accepted degradation do vary in other countries. Temocillin is an important addition to the anti-Gram-negative OPAT armamentarium and may reduce reliance on other broader spectrum, higher consequence agents.

